# Epstein–Barr virus positive diffuse large B‐cell lymphoma transformed into angioimmunoblastic T‐cell lymphoma after treatment

**DOI:** 10.1002/ccr3.4083

**Published:** 2021-05-05

**Authors:** Chaoyu Wang, Yi Gong, Qingming Jiang, Xiping Liang, Rui Chen

**Affiliations:** ^1^ Department of Hematology‐Oncology Chongqing Key Laboratory of Translational Research for Cancer Metastasis and Individualized Treatment Chongqing University Cancer Hospital Chongqing China; ^2^ Department of Pathology Chongqing Key Laboratory of Translational Research for Cancer Metastasis and Individualized Treatment Chongqing University Cancer Hospital Chongqing China

**Keywords:** angioimmunoblastic T‐cell lymphoma, diffuse large B‐cell lymphoma, Epstein–Barr virus

## Abstract

Angioimmunoblastic T‐cell lymphoma (AITL) is the subtype of mature T‐cell non‐Hodgkin lymphoma. Compared with diffuse large B‐cell lymphoma (DLBCL), AITL patients are frequently accompanied with Epstein–Barr virus (EBV) infection. To date, there is no report on the subsequent development of AITL in patients with EBV‐positive DLBCL. We performed a rare case of EBV‐positive AITL developing one year after initial diagnosis of EBV‐positive DLBCL. The patient showed poor response to the chemotherapy regimen, and poor survival.

## Graphical abstract

The left inguinal lymph node biopsy of initial diagnosis shows morphological (A, X200) and IHC staining with positive for CD20 (B, X200), PAX‐5 (C, X200), and Ki‐67 (D, X200).

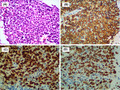

## 1 INTRODUCTION

To date, there is no report on the subsequent development of angioimmunoblastic T‐cell lymphoma (AITL) in patients with EBV‐positive diffuse large B‐cell lymphoma (DLBCL). We presented a rare case of EBV‐positive AITL developing one  year after initial diagnosis of EBV‐positive DLBCL. The patient showed poor response to the chemotherapy regimen and poor survival.

Angioimmunoblastic T‐cell lymphoma (AITL) is a subtype of mature T‐cell non‐Hodgkin lymphoma. Compared with diffuse large B‐cell lymphoma (DLBCL), AITL patients are frequently accompanied with Epstein–Barr virus (EBV) infection. To date, there is no report on the subsequent development of AITL in patients with EBV‐positive DLBCL. Here we described a rare case of EBV‐positive AITL developing one year after initial diagnosis of EBV‐positive DLBCL.

## 2 CASE DESCRIPTION

**FIGURE 1:**
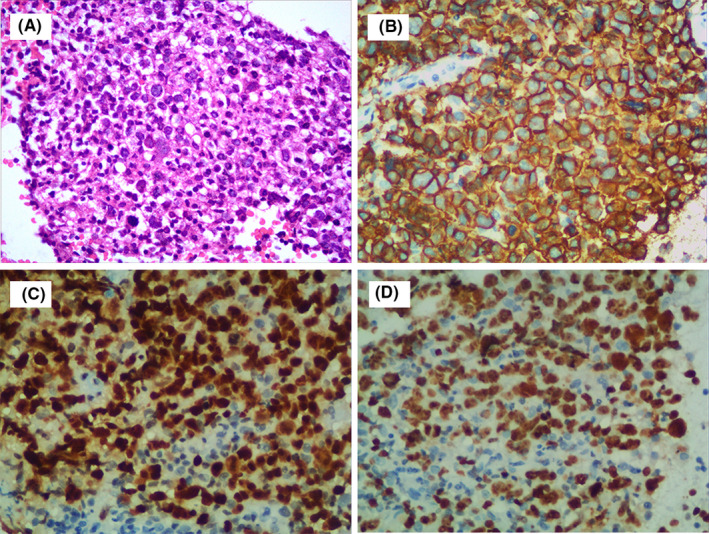
The left inguinal lymph node biopsy of initial diagnosis showing morphological (A, ×200) and immunohistochemistry staining with positive for CD20 (B, ×200), PAX‐5 (C, ×200), and Ki‐67(D, ×200)

In March 2019, an 83‐year‐old Chinese man was admitted to our hospital due to a month history of enlarged lymph nodes in the neck and bilateral inguinal region without pain and fever. Computed tomography (CT) scan disclosed generalized lymphadenopathy in the mediastinum, hilum, bilateral inguinal region, right lung, and right kidney. However, the complete blood count, coagulation markers, albumin, lactate dehydrogenase (LDH), creatinine, β_2_‐microglobulin, and alanine aminotransferase (ALT) were all within the normal range. The laboratory of EBV viral IgM‐capsid antigen (VCA) and EBV‐DNA was positive. The titer of EBV VCA was 1:80, and the copy number of EBV‐DNA was 1.06 × 10^6^/mL. Then, the patient underwent biopsy of left inguinal lymph node; a large number of diffuse large‐sized proliferation atypical lymphoid cells can be seen under microscope. Immunohistochemically (IHC), the atypical cells were positive for CD20, CD19, PAX‐5, and MUM‐1, but negative for CD3, CD5, CD10, Bcl‐6, CyclinD1, CD138, and TdT (Figure 1). C‐myc and Bcl‐2 were expressed by more than 60% and 20% of lymphoma cells. Ki67 was expressed by more than 80% of lymphoma cells. Besides, in situ hybridization for EBV‐encoded small RNA (EBER) staining was also positive. Bone marrow aspiration and trephine biopsy showed no infiltration of lymphoma cells. Based on the above, a pathological diagnosis of EBV‐positive DLBCL was made. After the diagnosis of EBV‐positive DLBCL, the patient received eight cycles of R‐miniCHOP (rituximab, cyclophosphamide, doxorubicin, vincristine, and prednisone) therapy, but his symptoms did not disappear and PET/CT scan showed no signs of complete remission (CR) or partially remission (PR) after treatment. The disease status was stable despite the immune‐chemotherapy administration.

**FIGURE 2:**
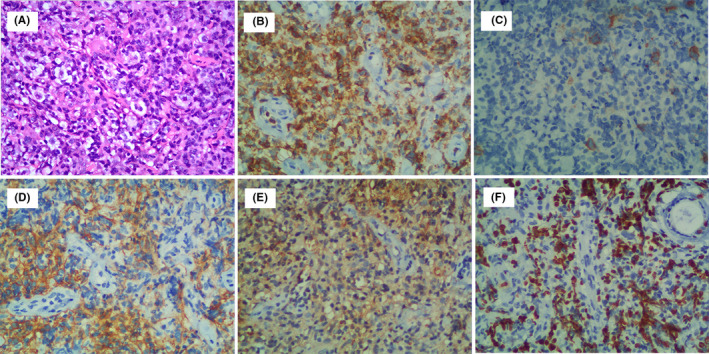
The biopsy of left inguinal lymph node 1 y after initial diagnosis showing morphological (A, ×200) and immunohistochemical finding with negative for CD20 (C, ×200), positive for CD5 (B, ×200), CD21(D, ×200), CXCL13 (E, ×200), Ki‐67 (F, ×200)

In March 2020, 1 year after initial diagnosis of EBV‐positive DLBCL, the patient was readmitted to our hospital because of nasopharyngeal discomfort. CT scan revealed multiple enlargements of mediastinum, hilum, bilateral inguinal lymph nodes, right lung, right kidney lymph nodes, and thickened nasopharyngeal wall. Bone marrow aspiration was normal. A left inguinal lymph node biopsy was performed, and to our surprise, it revealed angioimmunoblastic T‐cell lymphoma (AITL). Immunohistochemically, the cancer cells were positive for most pan‐T‐cell antigens such as CD3, CD4, CD5, CD7, and T follicular helper (TFH) biomarkers including CD10, Bcl‐6, and CXCL13, but negative for CD20, ALK, PD‐1, EMA, and Perforin (Figure 2). Ki67 was expressed in more than 45% of lymphoma cells. EBERs detection showed positive results. Molecular analysis was performed using formalin‐fixed, paraffin‐embedded tissue from the left inguinal lymph node biopsy specimen to assess the rearrangements of immunoglobulin heavy‐chain (IgH) gene, T‐cell receptor (TCR) gene by polymerase chain reaction (PCR). The case showed the presence of clonal TCR gene rearrangements, but no evidence of IgH gene rearrangements. A diagnosis of AITL secondary to EBV‐positive DLBCL was finally established. Since the patient showed poor response to the previous R‐miniCHOP treatment, new strategy of therapy should be considered. So, the histone deacetylase inhibitor chidamide and prednisolone were administered. The patient ultimately died of disease progression on July 3, 2020, after three months diagnosed with AITL. The overall survival time of this patient is fifteen months.

## 3 DISCUSSION

DLBCL is the most common type of malignant hematological diseases and also the most common type of B‐cell non‐Hodgkin lymphoma.^1^ Our previous retrospective study found that hepatitis B virus (HBV) is associated with aggressive B‐cell lymphoma, especially DLBCL.^2^ However, Epstein–Barr virus (EBV)‐associated DLBCL is relatively rare. AITL is the subtype of mature T‐cell non‐Hodgkin lymphoma. Compared with DLBCL, AITL patients are frequently accompanied with EBV infection, and some study found that EBV is involved in the occurrence and development of AITL.

EBV‐positive DLBCL patients following AITL have been reported in some case reports.^3^, ^4^, ^5^, ^6^ EBV infection may reduce the immune function of AITL patients and lead to B‐cell dysfunction. The occurrence of secondary DLBCL seems to be triggered by EBV infection. Some studies^7^, ^8^ have reported that AITL patients composite with DLBCL.

Only one case of AITL development after treatment of EBV‐negative DLBCL was reported.^9^ The patient received five cycles of R‐CHOP (rituximab, cyclophosphamide, doxorubicin, vincristine, and prednisone) chemotherapy regimen, and did not achieve any response. Six months after initial diagnosis of DLBCL, the patient developed AITL and ultimately died of progression disease after diagnosis of AITL two months. The overall survival is only eight months.

To our knowledge, there is no report on the subsequent development of AITL in patients with EBV‐positive DLBCL. Now, we presented a rare case of EBV‐positive AITL developing one year after initial diagnosis of EBV‐positive DLBCL. The patient showed poor response to the previous R‐miniCHOP chemotherapy regimen and then treated with chidamide and prednisolone. Unfortunately, similar to the above case, the patient finally died three months after diagnosis secondary EBV‐positive AITL. We hypothesized that EBV plays an important role in the development of DLBCL transform to AITL. In conclusion, this is a rare disease with a generally poor prognosis. However, the specific mechanism of this phenomenon is still unknown.

## ETHICS APPROVAL AND CONSENT TO PARTICIPATE

Written informed consent was obtained from all participants. Ethical approval was obtained from the Ethics Committee of the Chongqing Key Laboratory of Translational Research for Cancer Metastasis and Individualized Treatment, Chongqing University Cancer Hospital, Chongqing, China, in accordance with the ethical guidelines of the 1975 Declaration of Helsinki. Written informed consent was obtained from the patient for publication of this case report and any accompanying images. A copy of the written consent is available for review by the Editor‐in‐Chief of this journal.

## CONSENT FOR PUBLICATION

Written informed consent for publication was obtained from all participants.

## CONFLICT OF INTEREST

None declared.

## AUTHOR CONTRIBUTIONS

Chaoyu Wang, Yi Gong, Qingming Jiang, Xiping Liang, and Rui Chen conceptualized and designed the study. Chaoyu Wang and Yi Gong drafted the article. All the authors approved the final version.

## Data Availability

The data sets generated during and/or analyzed during the current study are available from the corresponding author upon reasonable request.
